# CUSTODIAL GRANDPARENTS: AN ANALYSIS OF PARENTING AND ITS IMPACT ON HEALTH

**DOI:** 10.1093/geroni/igz038.3282

**Published:** 2019-11-08

**Authors:** Karen C Clark, Kari R Lane, Linda Bullock

**Affiliations:** 1 Georgia State University, Atlanta, Georgia, United States; 2 University of Missouri, Columbia, Missouri, United States; 3 University of Missouri Columbia, Columbia, Missouri, United States

## Abstract

In the United States, there are 2.7 million grandparents raising grandchildren without a biological parent present (U.S. Census, 2014). Caring for grandchildren can present challenges and stressors to custodial grandparents as they find themselves adjusting to this unanticipated role. Despite the growing knowledge base related to custodial grandparents, there has been limited research into the relationship between parenting self-efficacy and psychological well-being. This study was guided by the Parenting Self-Efficacy Theory derived from Bandura’s Social Cognitive Theory. The purpose of this study was to examine the relationship between parenting self-efficacy and psychological well-being (anxiety and depression) among custodial grandmothers. Additionally, self-reported general health was examined to determine if it moderated the relationship between parenting self-efficacy and psychological well-being. Sixty-eight custodial grandmothers recruited across the United States participated in the study. Their mean age was 58 years old; 57% were Caucasian, 35% African American, and 3% Hispanic. With a mean income of $26,000.00, most were retired (32%) or working full-time (29%). Participants responded to psychometrically sound instruments measuring anxiety, depression, general health and parenting self-efficacy. Findings indicated parenting self-efficacy scores were not significantly associated with anxiety scores (r = .029; p = .816) or depression scores (r= -.207; p = .090) among participants. Furthermore, general health did not moderate a relationship between parenting self-efficacy, anxiety (R2= .030; p= .5753) or depression (R2= .051; p= .3376). Further research is needed to determine whether the role of parenting self-efficacy in custodial grandparents. Implications for policy will also be discussed

